# Harnessing the immune system in the treatment of cutaneous T cell lymphomas

**DOI:** 10.3389/fonc.2022.1071171

**Published:** 2023-01-12

**Authors:** Christopher J. Fay, Katherine C. Awh, Nicole R. LeBoeuf, Cecilia A. Larocca

**Affiliations:** Department of Dermatology, Center for Cutaneous Oncology, Brigham and Women's Hospital, Dana-Farber Cancer Institute, Harvard Medical School, Boston, MA, United States

**Keywords:** cutaneous T cell lymphoma, mycosis fungoides, immune system, skin neoplasm, oncology

## Abstract

Cutaneous T cell lymphomas are a rare subset of non-Hodgkin’s lymphomas with predilection for the skin with immunosuppressive effects that drive morbidity and mortality. We are now appreciating that suppression of the immune system is an important step in the progression of disease. It should come as no surprise that therapies historically and currently being used to treat these cancers have immune modulating functions that impact disease outcomes. By understanding the immune effects of our therapies, we may better develop new agents that target the immune system and improve combinatorial treatment strategies to limit morbidity and mortality of these cancers. The immune modulating effect of therapeutic drugs in use and under development for cutaneous T cell lymphomas will be reviewed.

## Capsule summary:

- Several therapeutic drugs in use and under development for cutaneous T cell lymphomas have immune modulatory effects
- Novel combinatorial treatment strategies have been developed based on the latest understanding of the favorable immune effects of individual therapies

## 1 Introduction

Cutaneous T cell lymphomas (CTCL) are a heterogeneous class of non-Hodgkin’s lymphomas that arise from skin tropic T cells, characterized by inflamed skin lesions with variable involvement of blood and lymph nodes. Mycosis fungoides (MF) and Sézary syndrome (SS) are the two most common clinical variants of CTCL with an incidence of approximately 10.2 per million persons in the United States (1). Patients with early stage MF (stage I-IIA) typically have an indolent disease course, but those with SS (Stage IV) have a 41% survival rate at five years (2).

The pathogenesis of CTCL remains unclear. Although environmental exposures and associations with infectious agents have been hypothesized, none have been definitively identified thus far (3). Furthermore, the oncogenic drivers of cutaneous T cell malignant transformation remain incompletely characterized given that genomic studies are largely derived from SS cohorts and the mutational landscape shows heterogeneous mutations and wide variation in chromosomal abnormalities (4). However, what is coming into focus is that the malignant T cell is a key driver of immunosuppression, reshaping the cutaneous microenvironment and broadly impairing cellular immunity, which is needed for an effective anti-tumor response (5). Please see Krejsgaard et al. for a comprehensive review of “malignant inflammation”—describing the mechanism by which malignant T cells remodel the immune environment from early to advanced disease via release of immunosuppressive cytokines, modulation of chemokines, and engagement with stromal and immune cells (6). In addition to directly affecting immune cell function, the malignant T cells can also impair the epidermal skin barrier (7). This allows bacteria such as *Staphylococcus aureus* to further influence the tumor microenvironment (TME). *S. aureus, *via release of enterotoxins, pore-forming α-toxins, and lipoproteins, suppresses anti-tumor immunity. Staphylococcal enterotoxins function as superantigens activating malignant T cells and inducing an immunosuppressive phenotype, characterized by upregulation of CD25, FOXP3, IL-17, and miR-155 expression (8). Staphylococcal α-toxin more easily causes apoptosis in benign CD4+ and CD8+ T cells and can interfere with the cytotoxic killing capacity of anti-tumor CD8+ T cells (8, 9). And lastly, bacterial lipoproteins promote a type 2 helper T cell (T_h_2)dominant milieu by activating toll-like receptors on keratinocytes to release pro-tumorigenic cytokines (8). In fact, use of antibiotics in patients with CTCL has led to remarkable clinical responses (9), a unique example of how targeting drivers of pro-tumorigenic inflammation can improve disease outcomes.

The immunosuppressive effects of malignant T cells can be reversed with skin-directed and systemic therapies (10–12). However, it is unclear whether this is mediated solely by depletion of malignant T cells or if therapies that independently augment the immune system toward a type 1 helper T cell (T_h_1) bias have greater efficacy. By understanding how past and emerging therapies can modulate the immune system we hope more effective multimodal and combination therapies will be developed.

**Figure 1:**
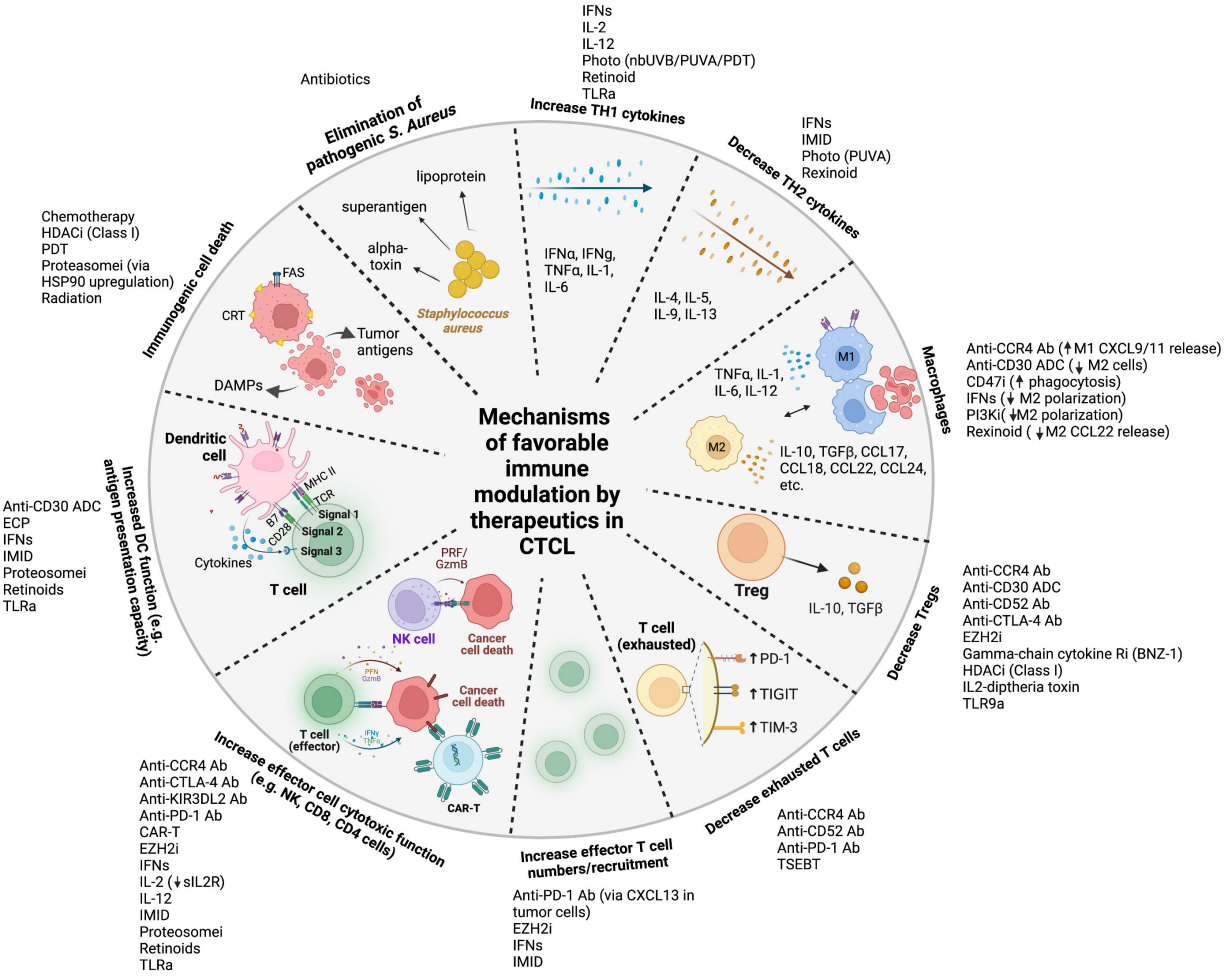
Mechanisms of favorable immune modulation by therapeutics in cutaneous T cell lymphoma Legend: ab, antibody; ADC, antibody-drug conjugate; anti-CTLA-4, anti-cytotoxic T-lymphocyte-associated protein 4; anti-PD-1, anti-programmed cell death protein 1; CAR, chimeric antigen receptor; CCR4, anti-C-C chemokine receptor 4; CD47i, cluster of differentiation 47 inhibitor; CRT, calreticulin; DAMPs, damage associated molecular patterns DC, dendritic cell; ECP, extracorporeal photopheresis; EZH2i, enhancer of zeste homolog 2 inhibitor; GzmB, granzyme B; HDACi, histone deacetylase inhibitor; IFNs, interferons; IL, interleukin; IMID, immunomodulator (lenalidomide); MHC, major histocompatibility complex; nbUVB, narrowband ultraviolet B; NK, natural killer; PI3Ki, phosphoinositide 3-kinase inhibitor; PRF, perforin; proteosomei, proteosome inhibitor; PUVA, psoralen plus ultraviolet A; sIL2R, soluble interleukin-2 receptor; TCR, T cell receptor; TGF, transforming growth factor; T_h_1, Type 1 T helper; T_h_2, Type 2 T helper; TIGIT, T cell immunoreceptor with Ig and ITIM domains; TIM-3, T cell immunoglobulin and mucin-domain containing-3; TLRa, toll like receptor agonist; TNF, tumor necrosis factor; TSEBT, total skin electron beam therapy.

We aim to provide a comprehensive and updated review of the immune modulating effects of all classes of therapeutics used and under development for CTCL (13). We caution that many drugs are incompletely understood in terms of their mechanism of cell death, direct impact on tumor cell function, and effect on different aspects of the immune system. Here, we report on the broadly accepted mechanism of action for each drug and primarily focus on the effect of treatment on the immune environment. In addition, the mechanistic rational for several combination therapies under investigation will be summarized. Favorable (**Figure 1**) and unfavorable immune modulatory effects are summarized in **Table 1**. Please see Garcia-Diaz et al. for a comprehensive review integrating how different signaling pathways targeted in CTCL interact, which is beyond the scope of the review (14).

**Table 1:** Favorable and unfavorable immune modulatory effects of CTCL therapies.

Drug	Favorable immune modulatory effect	Unfavorable immune modulatory effect
**Alkylating Agents** *Chlorambucil*, *Mechlorethamine*, *Cyclophosphamide*	• Hypersensitivity reaction increase T_h_1 cytokines • Immunogenic cell death (cyclophosphamide)	• Myelosuppression
**Anti-Metabolites** *Methotrexate*, *Pralatrexate*	• Pro-apoptotic • Potent inhibitor of JAK-STAT (methotrexate)	• Myelosuppression • Increases adenosine levels (broad immunosuppressive function on DC maturation, effector T cell and NK cell function, increases M2 macrophage polarization)
**Anthracyclines** *Pegylated liposomal doxorubicin*	• Immunogenic cell death	• Myelosuppression
**BCL-2 inhibitor** *Venetoclax*	• Pro-apoptotic • Spares antigen-stimulated effector T cells	• Neutropenia • Lymphopenia • Leukopenia • Decreases naïve T cells
**CAR T cell therapy** *ATLCAR.CD30 cells*, *LB1901*, *CD37 directed CAR T cells*	• Increases cytotoxicity, activates cytokine-driven signaling, recruits innate immune cells, and promotes T cell activation (4^th^ and 5^th^ generation CARs) • Higher affinity than conventional immune processes for binding unprocessed target antigens (including major histocompatibility complex-independent forms)	• May kill healthy T cells, possibly prompting T cell aplasia and immunodeficiency • May promote tumor outgrowth after *ex vivo* amplification and resistance • May promote self-killing of CAR-T cells (“fratricide”)
**CCR4 inhibitor** *Mogamulizumab*	• Preferentially depletes CCR4+ T cells, including Tregs and exhausted T cells (often CCR4+) • Increases NK cell killing function (defucosylation of Fc portion) • Pro-inflammatory tumor associated macrophages in MAR	• Reduces clearance of resident memory T cells in skin (including malignant T cells) • Lymphopenia
**CD30 antibody-drug conjugate** *Brentuximab vedotin*	• Pro-apoptotic • May deplete immunosuppressive M2 type tumor associated macrophages • May deplete CD30+ Tregs but not CD30+CD8+ T cells • Induces DC maturation	• Myelosuppression
**CD47 inhibitor** *TTI-621(Fc region of IgG1 with CD47 binding domain of SIRPa)*	• Impairs antiphagocytic signals • Promotes phagocytic signals	
**CD52 inhibitor** *Alemtuzumab*	• Elimination of Tregs, exhausted T cells, and malignant T cells	• Reduces clearance of resident memory T cells in skin (including malignant T cells) • Eliminates innate and adaptive immune cells (including effector cell populations)
**EZH2 inhibitor** *Valemetostat*	• Decreases Tregs • Improves NK cell function	• Myelosuppression • Impairs effector T cell function • Impairs DC antigen presentation
**γ-chain cytokine inhibitor** (selective for IL-2, IL-9, IL-15) *BNZ-1*	• Impair cytokine-driven tumor cell survival • Decreases Treg activity	• Impairs cytokine-driven effector T cell survival
**HDAC inhibitors** *Romidepsin*, *Belinostat*, *Panobinostat*, *Resminostat*, *SHP-141*, *Vorinostat*	• Decreases Tregs (Class I HDACi) • Increases immunologic cell death	• Suppresses NK cell and DC function • Increases Tregs (panHDACi)
**IL-2-diptheria toxin fusion protein** *E7777*	• Pro-apoptotic • Depletes Tregs	• Only targets activated T cells (resting Tregs remain in circulation) • Impairs DC maturation
**IL-2**	• Growth and differentiation of T cells into memory producing anti-tumor T cells • Increases T_h_1 cytokines • Overcomes immunosuppressive sIL2R levels	• Induces and maintains Tregs
**IL-12**	• Increases T_h_1 cytokines • Promotes NK cell and cytotoxic T cell activity	• Potential for rapid development of lethal inflammatory syndrome
**Immunomodulator (Thalidomide derivative)** *Lenalidamide*	• Increases antigen presentation • Increases CD8+ T cell activity • Decreases T_h_2 cytokine expression	• Increases Tregs
**Immune checkpoint (PD-1, PD-L1, CTLA-4) inhibitors** *Durvalumab*, *Nivolumab*, *Pembrolizumab*, *Tremelimumab*, *Ipilimumab*	• Expands cytotoxic CD4+ and CD8+ T cells (no longer exhausted T cells) • Depletes Tregs (CTLA-4)	• Risk of hyperprogression (PD-1 has been shown to act as a tumor suppressor) • Increases Tregs? (CTLA-4)
**Interferons (Type I and II)** *IFN-a2a*, *IFN a2b*, *IFN-γ*	• Anti-proliferative and pro-apoptotic effects • Increases T_h_1 cytokines and decreases T_h_2 cytokines • Increases antigen presentation • Stimulates NK cell activity • Recruits NK cells and T_h_1 cells (increases CXCL10/11) • Decreases recruitment of Tregs (decreases CCL17)	• IFN-γ induces PD-L1 in tumor tissue
**JAK inhibitors** *Cerdulatinib*, *Ruxolitinib*	• Combats frequently dysregulated JAK-STAT pathway that may be increasing survival and resistance to apoptosis in malignant T cells	• May suppress interferon-associated favorable effects in effector T cells
**KIR3DL2 inhibitor** *Lacutamab*	• Pro-apoptotic (via ADCC)	• Lymphopenia
**PI3K inhibitors** *Duvelisib*, *Tenalisib*	• Pro-apoptotic • Promotes CD8+ T cell activation and cytotoxicity • May deplete immunosuppressive M2 type and increase M1 type tumor associated macrophages	• Decreases migration of effector T cells • Decreases immune synapse formation with APCs
**Proteosome inhibitor** *Bortezomib*	• Pro-apoptotic and anti-proliferative effects • Increases dendritic cell and NK cell activity (possibly *via* immunogenic cell death)	• Impairs antibody-mediated responses by enhanced B cell apoptosis
**Radiation (ionizing)** *LEBT*, *TSEBT*, *brachytherapy*	• Direct pro-apoptotic effects on malignant T cells • Induces immunogenic cell death • Increases MHC class I on tumor cells • Decreases blood exhausted T cells and increases IFN-γ secretion in PBMCs of SS patients (TSEBT)	• Upregulation of PD-L1 on tumor cells (immune evasion)
**Radiation (non-ionizing)** *nbUVB*, *PUVA*, *ECP*, *PDT*	• Direct pro-apoptotic effects on malignant T cells (all modalities) and keratinocytes (nbUVB, PUVA) • Increases T_h_1 cytokines (nbUVB) • Increases T_h_1 cytokines and decrease T_h_2 cytokines (PUVA) • Turnover from T_h_2 to T_h_1 T cells (PUVA) • Generation of anti-tumor T cells (PUVA) • Maturation of DC (ECP) • Decreases sILR levels (ECP) • Immunogenic cell death (PDT) • Increases T_h_1 cytokines (PDT) • Preferential apoptosis of malignant over benign T cell (PDT)	• Decreases antigen presentation (nbUVB) • Decreases NK cell activity (nbUVB) • Increases Tregs (nbUVB, ECP) • Increases T_h_2 cytokines (nbUVB) • Effector T cells more susceptible to ROS than Tregs (PDT) • ROS may cause hyporesponsiveness of effector T cells and NK cells (PDT)
**Retinoids** *Acitretin*, *isotretinoin*	• Direct apoptotic effect • Induces T_h_1 cytokines • Increases antigen presentation • Increases NK cell activity	
**Rexinoids** *Bexarotene*	• Anti-proliferative, pro-apoptotic effects • Downregulates T_h_2 cytokines • Decreases CCL22 by M2 tumor associated macrophages (recruits Tregs)	• Neutropenia
**TLR agonists** *Imiquimod*, *Resiquimod*, *CPG-7907*, *Poly-ICLC*	• Maturation of dendritic cells and increased antigen presentation • Increases IFN-α, IL-12 • Stimulates of NK cells	• Myelosuppression

BCL, B cell lymphoma; CAR, chimeric antigen receptor; CD, cluster of differentiation; CTLA-4, cytotoxic T-lymphocyte associated protein 4; DC, dendritic cell; EZH, enhancer of zeste homolog; HDAC, histone deacetylase; IFN, interferon; IL, interleukin; JAK-STAT, Janus kinase-signal transducer and activator of transcription; MAR, mogamulizumab associated rash; MMAE, monomethyl auristatin E; NK cell, natural killer cell; PD-1, programmed cell death protein 1; PD-L1, programmed death-ligand 1; PI3K, phosphoinositide 3-kinases; ROS, reactive oxygen species; T_h_1, type 1 helper T cell; T_h_2, type 2 helper T cell; TLR, toll-like receptor; Treg, regulatory T cell.

A summary of pivotal clinical trials along with their response rates for all reviewed therapies are included in **Tables 2**–**4** to help put into context the potential importance of observed immune modulatory effects. Notable responses are listed in the text. Unless otherwise stated the response rate (RR) is determined by the sum of the rates of complete and partial responders. Of note, definitions of complete and partial response vary within each study until the establishment of response criteria for MF/SS by Olsen et al. published in 2011 (56). In general, for assessment of skin response, a complete response (CR) requires 100% clearing of skin disease and a partial response (PR) requires ≥ 50% reduction in skin tumor burden as determined by the severity weight assessment tool (SWAT) (57) or its modification (mSWAT) (44) score compared with baseline, sustained for 4 weeks. Prior studies were based on change in body surface area affected. Later studies required global responses in the cutaneous and extracutaneous compartments, including blood, lymph node and viscera, as evaluated by CT scans and peripheral lymphocyte counts (56).

**Table 2:** Biologic response modifiers, cellular immunotherapy, immune checkpoint inhibitors, and toll-like receptor agonists for the treatment of CTCL.

Reference	Drug	Mechanism of action	Route	Study design	Disease stage	Patients (N)	Response rate	Disease outcome
**BIOLOGIC RESPONSE MODIFIERS**								
Olsen et al. (1989) (14)	IFN-α	Type I interferon	SC	Controlled trial	IA-IVA MF and SS	22	64%; CR 27%	4-27.5 months
Kaplan et al. (1990) (15)	IFN-γ	Type I interferon	SC	Phase II trial	CTCL with at least one prior therapy	16	31%	mPFS 10 months
Querfeld et al. (2007) (16)	IL-2	Cytokine	SC	Phase II trial	IA-IVB MF and SS	22	18%	mDOR 3 months
Duvic et al. (2006) (17)	IL-12	Cytokine	SC	Open label phase II trial	IA-IIA MF with >3 prior therapies	23	43%	mDOR 3 months
**CELLULAR THERAPY**								
Wu et al. (2009) (18)	Allogenic SCT	Replace bone marrow with donor healthy stem cells	IV	Meta-analysis	MF and SS, mostly stage IVA	20	20% mortality	OS 85% at 1 year and 80% at 5 years
Wu et al. (2009) (18)	Autologous SCT	Replace bone marrow with own healthy stem cells	IV	Meta-analysis	MF and SS, mostly stage IIB	19	53% mortality	OS 68% at 1 year and 23% at 5 years
**IMMUNE CHECKPOINT INHIBITORS**								
Lesokhin et al. (2016) (19)	Nivolumab	PD-1 inhibitor	IV	Open-label phase I trial	MF	13	15%	mDOR NR
Khodadoust et al. (2020) (20)	Pembrolizumab	PD-1 inhibitor	IV	Single-arm phase II trial	Stage IIB-IV MF and SS	24	38%; CR 8%	mDOR NR
Querfeld et al. (2019) (21)	Durvalumab (with lenalidomide)	PD-L1 inhibitor	IV	Randomized phase I/II trial	Relapsed or refractory CTCL	9	78%	mDOR of 6 months
Querfeld et al. (2017) (22)	TTI-621	CD47 inhibitor (ADCP)	SC	Phase I open label trial complete	Relapsed or refractory MF and SS	6	Not available, one CR	Not available
**TOLL-LIKE RECEPTOR AGONISTS**								
Chong et al. (2004) (23)	Imiquimod cream	TLR7 agonist	Topical	Double-blind placebo-controlled pilot study	Early stable patch or plaque stage MF	4	Mean decrease in SA 8.9%	Not available
Rook et al. (2015) (10)	Resiquimod gel	TLR7/8 agonist	Topical	Phase I trial	IA-IIA CTCL	12	75%; CR 30%	Not available
Kim et al. (2010) (24)	CPG-7909	TLR9 agonist	SC	Phase I trial	Refractory stage IB to IVA CTCL	28	32%; CR 11%	>24 weeks

CD, cluster of differentiation; CR, complete remission; CTCL, cutaneous T cell lymphoma; IFN, interferon; IL, interleukin; IV, intravenous; mDOR, median duration of response; MF, mycosis fungoides; mPFS, median progression-free survival; NR, not reached; OS, overall survival; PD-1, programmed cell death protein; PD-L1, programmed death-ligand 1; SA, surface area; SC, subcutaneous injection; SCT, stem cell transplantation; SS, Sézary syndrome; TLR, toll-like receptor.

**Table 3:** Chemotherapies and radiotherapies for the treatment of CTCL.

**CHEMOTHERAPIES**												
Coors et al. (2000) (25)	Chlorambucil (with fluocortolone)		Alkylating agent	PO		Uncontrolled pilot study	Erythrodermic CTCL (stages III-IVb)		13	100%; CR 54%		mDOR 12 months
Quereux et al. (2008) (26)	Doxorubicin		DNA intercalation	IV		Prospective open trial	II-IV CTCL		25	56%; CR 20%		mPFS 5 months
Lessin et al. (2013) (27)	Mechlorethamine gel*		Alkylating agent	Topical		Randomized, observer-blinded trial	IA-IIA		260	58.5%		mDOR NR, > 10 months
Zackheim et al. (1996) (27)	Methotrexate		Dihydrofolate reductase inhibitor	PO or SC		Retrospective study	Erythrodermic CTCL		29	58%; CR 41%		mDOR 31 months
Horwitz et al. (2012) (28)	Pralatrexate		Dihydrofolate reductase inhibitor	IV		Dose-escalation study	MF, SS, or pcALCL		29	45%; CR 3%		mDOR NR, >6 months
**IONIZING RADIATION**												
Wilson et al. (1998) (29)	Local radiation	Electrons, median 20 Gy		Radiation	Retrospective cohort		MF		21 patients (32 sites)		CR 97% of lesions	<1% relapse
Thomas et al. (2013) (30)	Local radiation	Electrons, single dose of equal to or greater than 7Gy		Radiation	Retrospective cohort		MF		58 patients (270 lesions)		CR 94% of lesions	1% relapse at 41.8 months
Hoppe et al. (2015) (31)	TSEBT	Electrons, 12 Gy		Radiation	Phase II, 3 studies pooled		MF		33		88%; CR 27%	mDOR 70.7 weeks
Navi et al. (2011) (32)	TSEBT	Electrons, 36Gy (range 30-40 Gy)		Radiation	Retrospective cohort		MF		180		100%; CR 60%	mDOR 29 months (T2); mDOR 9 months (T3)
Goddard et al. (2015) (33)	Brachytherapy	Gamma radiation; high dose rate		Catheter radiation	Case series		Acral CTCL lesions		6		100%	mDOR NR, >15.8 months
**NON-IONIZING RADIATION**												
Gathers et al. (2002) (34)	Narrowband UVB (nbUVB)	Phototherapy		UV light	Retrospective cohort		MF stage IA and IB	24			83%; CR 29%	mDOR 12.5 weeks
Ponte et al. (2010) (35)	Psoralen-UV-A (PUVA)	Phototherapy		PO + UV light	Retrospective cohort		MF stage IA, IB, IIA	95			87.4%; CR 62%	mPFS 11.5 months
Talpur et al. (2011) (36)	ECP	8-MOP + UV-A light		SC or PO + UV light	Open label pilot trial		MF stage IA-IIA	19			42%; CR 5%	mDOR 6.5 months
Gao et al. (2019) (37)	ECP	8-MOP + UV-A light		PO + UVA light	Retrospective cohort		SS, E-MF	65			69% skin response; CR 6% (skin, blood, node, viscera)	Median time on ECP 17 months
Kim et al. (2022) (38)	Topical hypericin	Photodynamic therapy (PDT)		Visible light + topical	Phase III trial		Stage IA-IIA MF	116			ILRR 49% after 3 cycles; CR 13%	mDOR 7.5 months

CR, complete remission; CTCL, cutaneous T cell lymphoma; ECP, extracorporeal photopheresis; E-MF, erythrodermic mycosis fungoides; ILRR, index lesion response rate; IV, intravenous; mDOR, median duration of response; MF, mycosis fungoides; mPFS, median progression-free survival; MOP, methoxypsoralen; NR, not reached; pcALCL, primary cutaneous anaplastic large cell lymphoma; PO, oral; PUVA, psoralen + ultraviolet light A; SC, subcutaneous injection; SS, Sézary syndrome; TSEBT, total skin electron beam therapy.

*FDA approved for CTCL.

**Table 4:** Targeted therapies for the treatment of CTCL.

Reference	Drug	Mechanism of action	Route	Study design	Disease stage	Patients (N)	Response rate	Disease outcome
**FUSION PEPTIDES/PROTEINS**								
Querfeld et al. (2020) (39)	BNZ-1	IL-2, IL-9, and IL-15 inhibiting peptide gamma	IV	Open label phase I/II trial	Treatment refractory MF and SS	19	63%; CR 5%	mDOR 9.2 months
Kawai et al. (2021) (40)	E7777 (reformulated Denileukin diftitox)	IL-2 -diphtheria toxin	IV	Phase II-III trial	Relapsed or refractory CTCL, Japanese cohort	37	32%	mPFS 4.2 months
**ANTIBODIES**								
Lundin et al. (2003) (41)	Alemtuzumab	CD52 inhibitor	IV	Phase II trial	Stage II-IV MF and SS	22	55%; CR 32%	mPFS 12 months
Prince et al. (2017) (42)	Brentuximab vedotin*	CD30 antibody-drug conjugate	IV	Randomized phase II trial	CD30+ MF or pcALCL	64	56.3%	mPFS 16.7 months
Bagot et al. (2019) (43)	Lacutamab (IPH4102)	KIR3DL2 inhibitor	IV	Open-label phase I trial	Relapsed or refractory MF and SS	44	36%	mDOR NR
Kim et al. (2018) (44)	Mogamulizumab*	CCR4 inhibitor	IV	Randomized phase III trial	Stage IB-IV MF and SS	372	35%	mPFS 7.7 months
**SMALL MOLECULES**								
Duvic et al. (2001) (45)	Bexarotene*	Retinoid X receptor agonist	PO	Open label phase II trial	Stage IIB-IV	94	45%; CR 15%	mDOR 6 months
Zinzani et al. (2007) (46)	Bortezomib	Proteosome inhibitor	SC or IV	Phase II trial	Relapsed or refractory MF	10	70%; CR 10%	mDOR 7 months
Horwitz et al. (2018) (47)	Cerdulatinib	Dual SYK/JAK inhibitor	PO	Phase II trial	Refractory CTCL	37	35%	mDOR NR (>185 days);
Horwitz et al. (2018) (48)	Duvelisib	PI3K-δ,γ inhibitor	PO	Phase I trial	Relapsed or refractory CTCL	19	32%	mPFS 4.5 months
Querfeld et al. (2014) (49)	Lenalidomide	Thalidomide derivative	PO	Open-label phase II trial	Refractory MF and SS	32	28%	mDOR 10 months
Duvic et al. (2013) (50)	Panobinostat	panHDAC inhibitor	PO	Phase II trial	MF and SS	60	20%	mPFS 3.7 months
Whittaker et al. (2010) (51)	Romidepsin*	Class I HDAC inhibitor	IV	Open-label, phase I trial	Stage IB-IVA pretreated	96	30%; CR 6%	mDOR 15 months
Moskowitz et al. (2021) (52)	Ruxolitinib	JAK1/2 inhibitor	PO	Phase II trial	MF	5	20%	>18 months
Huen et al. (2020) (53)	Tenalisib	PI3K-δ,γ inhibitor	PO	Phase I/Ib trial	Relapsed or refractory CTCL	20	45%	mDOR 3.8 months
Olsen et al. (2007) (54)	Vorinostat	PanHDAC inhibitor	PO	Phase IIb trial	Refractory stage IB-IVA MF or SS	74	29.7%	mDOR NR, > 185 days

CR, complete remission; CTCL, cutaneous T cell lymphoma; HDAC, histone deacetylase; IL, interleukin; IV, intravenous; JAK-STAT, Janus kinase-signal transducer and activator of transcription; mDOR, median duration of response; MF, mycosis fungoides; mPFS, median progression-free survival; NR, not reached; pcALCL, primary cutaneous anaplastic large cell lymphoma; PI3K-δ,γ, phosphatidylinositol 3-kinase delta and gamma; PO, oral; SC, subcutaneous injection; SS, Sézary syndrome; SYK, spleen tyrosine kinase.

*FDA approved for CTCL.

## 2 Biologic response modifiers

The first reported use of immune stimulating agents for the treatment of cancer was in 1891 by Coley, who used bacterial cell extracts to treat osteosarcoma (58). Since the discovery of the first interferon in 1957 and its later isolation in sufficient quantities from donated blood for treatment in the 1970s, interferon alpha (IFN-α) and its other subtypes (β and γ) have been tried in numerous cancers (58). It was quickly noted that lymphomas, including MF, were among the most responsive to this treatment modality (58).

### 2.1 Interferons

Interferons are pleiotropic cytokines with diverse effects on cellular function and immune response. There are three types of interferons, I (IFN-α and IFN−β), type II (IFN-γ), and type III interferons (IFN-λ). Type I and II interferons primarily activate the Janus kinase/signal transducer and activator of transcription (JAK-STAT) pathway to induce expression of interferon responsive genes by binding to IFN-response elements (59, 60). A variety of STAT dimers may be activated by these interferons, however the mechanisms driving particular STAT usage and specificity are not understood (61). These interferons have also been shown to activate the mitogen-activated protein kinase (MAPK), phosphoinositide 3-kinase (PI3K) and mammalian target of rapamycin (mTOR) signaling pathways, independent of STAT signaling. Surprisingly, activating mutations and signaling in these aforementioned pathways have all been implicated in the pathogenesis of MF/SS (62). Therefore, the cellular context and concurrent activation or absence of activation of other IFN-dependent signaling pathways may drive divergent biologic responses (e.g. mitogenic stimuli versus induction of apoptosis) as interferons have not been seen in practice to promote disease progression.

Only type I and II interferons have been used as cancer therapies. They exhibit direct anti-proliferative and pro-apoptotic effects on malignant cells (59, 60). Immunomodulating effects of interferons include an increase in T_h_1 cytokines and a decrease in T_h_2 cytokines (59). They have been shown to boost the anti-tumor immune response by activating dendritic cells to enhance tumor antigen presentation to CD4+ helper and CD8+ cytotoxic T cells and stimulate NK cell activity *in vivo* (59, 63). After IFN-α2a and IFN-γ treatment, there is a decrease in chemokine receptor ligands (CCLs) CCL17 and CCL18 and an increase in C-X-C motif chemokine ligands (CXCLs) CXCL10 and CXCL11 by M2 macrophages (64). This leads to a favorable TME as CCL17 is important for recruitment of T regulatory (Treg) cells and CXCL10/CXCL11 recruits NK cells and activated T_h_1 cells (65–67). In CTCL, interferon α2a, α2b, and γ are used as a monotherapy or in combination with numerous other therapies (**Table 2**) (15, 60, 68). SS patients treated with IFN-α have decreased IL-4 and IL-5 expression, cytokines that promote T_h_2 polarization (69). Interestingly, compared to chemotherapy-based regimens, IFN-α can induces longer-term remissions (70).

Some patients have an initial or acquired resistance to interferon therapy (60). The development of neutralizing antibodies, mutations in the JAK-STAT pathway, down-regulation of IFN receptors, deregulation of cell cycle control, apoptosis and signal transduction may explain IFN-α resistance (60, 71–74). Therefore, the malignant T cells largely develop intrinsic mechanisms of resistance to biologic response modifiers that are not overcome by favorable effects on the immune microenvironment. However, patients resistant to IFN-α may respond to interferon-γ (16).

### 2.2 Interleukin-2

IL-2 was first used in cancer immunotherapy in 1984 for the treatment of melanoma (75). IL-2 is secreted primarily by antigen stimulated CD4+ T cells, as well as by CD8+ T cells, NK cells, and activated dendritic cells (75). The desirable effects of IL-2 are the stimulation of growth and differentiation of T cells into memory producing anti-tumor T cells (75). It has been shown to increase release of IFN-α, TNF-α, IL-1, IL-5, and IL-6 (60). Furthermore, recombinant IL-2 can be used to overcome elevated levels of soluble IL-2 receptor (IL-2R) (76), which acts as an inhibitor of IL-2 and inhibits NK cell activity *in vitro* (77). However, IL-2, *via* a JAK-STAT3 pathway, can also induce and maintain Tregs which are characterized by their expression of the master transcription factor FOXP3 and their ability to suppress other T cells *via* both cell-contact dependent and independent mechanisms (5, 75). A study of IL-2 in MF/SS patients showed only an 18% partial response rate and mixed immune responses (17). A subset of patients treated with IL-2 had an expansion of CD25+ cells in CD3+CD4+ populations, but it was unclear whether this was an expansion of Tregs, malignant T cells or activated anti-tumor T cells.

### 2.3 Interleukin-12

IL-12 has also been investigated in MF in an open-label trial (**Table 2**) (23). IL-12 has been shown to increase the T_h_1 cytokine IFN-γ and promote NK cell and cytotoxic T cell activity (23, 78, 79). However, this recombinant protein is not readily available for clinical practice (68) and earlier studies showed high rates of toxicity (80). An intratumorally delivered DNA plasmid expressing IL-12 was also being evaluated in MF, but the trial was terminated due to company resource constraints (clinicaltrials.gov identifier NCT01579318).

## 3 Cellular immunotherapy

### 3.1 Chimeric antigen receptor T cell therapy

CARs are recombinant receptor proteins engineered to redirect the function of T lymphocytes (81). They are composed of an extracellular antigen binding moiety, extracellular hinge region, transmembrane domain, and intracellular T cell activation component (82). The CAR is anchored to the plasma membrane by the transmembrane domain, which fosters stability of antigen binding. The extracellular antigen binding moiety is linked to the intracellular signaling domain: upon CAR-antigen binding, the cell is activated and triggers cytotoxicity towards the targeted cancer cells. Tumor cells attempt to evade the immune system through a variety of mechanisms, such as decreased expression of major histocompatibility complex (MHC) class I molecules, which reduces the antigen presentation capacity (83). CAR T cells have many advantages in comparison to conventional immune approaches, including higher antigen affinity for binding unprocessed target antigens independent of MHC (82). Additionally, with their own costimulatory domains, CAR T cells can increase immune responses to apoptosis and lyse target cells (82). Recent advances have led to fourth and fifth generation CARs, which have a transgenic cytokine or intracellular domain of a cytokine receptor to increase cytotoxicity, activate cytokine-driven signaling, recruit innate immune cells for antigen-negative tumor cells, and promote T cell activation (84, 85). This therapy has been approved for and is successful in B cell hematopoietic tumors, among others.

Use of CAR T therapy for CTCL, however, has specific challenges. First, as healthy and malignant T cells share commonalities, this could inadvertently lead to CAR T cells killing healthy T cells, prompting T cell aplasia and immunodeficiency (82). There is no treatment for quickly remedying T cell depletion, unlike intravenous immunoglobulin for B cell aplasia, which makes this a more concerning adverse event in CTCL patients. Additionally, both healthy T cells and circulating tumor T cells may be simultaneously collected when the CAR product is being created. This contamination may promote both tumor outgrowth after *ex vivo* amplification and resistance (82, 86). In a patient with B-ALL, for instance, the CAR gene was accidentally introduced into a leukemic B cell during the T cell manufacturing which can induce resistance (87). With this kind of contamination occurring in CTCL, there can be an interaction between the “CAR tumor T cells” and the tumor antigen on their own surfaces. This creates a “loop situation” that hides the surface antigen, and the activity of the non-contaminated CAR T cells is hindered. Another possible complication of CAR T cell therapy in CTCL is fratricide, which is self-killing of CAR T cells when the target antigen is common between malignant T cells and T effector cells (82). To et al. has proposed ways to minimize these risks of CAR T cell therapy in CTCL, although it is clear that CTCL presents more obstacles than B cell tumors for application of this therapy (82). CAR T cell therapy in preclinical *in vivo* and *in vitro* studies has been promising, demonstrating anti-tumor activity, without apparent off-target effects (88). To date there are three ongoing phase I trials investigating the safety and efficacy of CAR T cell therapy in CTCL. One clinical trial targets CD30 (clinicaltrials.gov identifiers: NCT03602157). In this same trial, some participants will receive CAR T therapy that targets CD30 but also has been genetically modified to express CCR4, which is important in trafficking cells to the skin. Another clinical trial involves a CAR T named LB1901 that targets CD4 (clinicaltrials.gov NCT04712864). Lastly, there is a clinical trial for a CAR T that targets CD37 (clinicaltrials.gov NCT04136275).

### 3.2 Hematopoietic stem cell transplantation

Hematopoietic stem-cell transplantations may be autologous, which involves the patient’s own stem cells, or allogenic, which involves donor stem cells. Allogenic stem cell transplantation (alloSCT) was first performed in CTCL patients in the 1990s with hope of a potential cure (89). While no randomized controlled trials comparing the efficacy of alloSCT with conventional therapy in advanced CTCL have been performed (90), retrospective analyses have demonstrated long-term remission in advanced stage CTCL after alloSCT (91–94). It is worth mentioning that compared to alloSCT, autologous stem cell transplant (autoSCT) has significantly lower overall survival and shorter event-free survival, failing to show durable responses with progression noted as early as 100 days (18, 95). Interestingly, patients with alloSCT have shown multiple pieces of evidence supporting a “graft-versus-lymphoma/graft-versus-tumor” (GvL/GvT) effect thought to be mediated by donor T cells and NK cells: there is durable remission associated with chronic graft-versus-host disease, relapse or progression may be triggered with initiation of immunosuppressive treatment, and this relapse or progression upon initiation of immunosuppressive treatment responds to discontinuation of the drugs and to infusion of donor lymphocytes (95, 96). The success of alloSCT and not autoSCT to induce long-term remissions is the most striking evidence yet that by harnessing the immune system we may achieve our greatest success in the treatment of CTCL.

## 4 Chemotherapy

Several chemotherapeutic agents are recognized by the National Comprehensive Cancer Network for the treatment of MF/SS. These drugs include dihydrofolate reductase inhibitors (methotrexate and pralatrexate), anthracycline topoisomerase inhibitors (doxorubicin), nucleosides (gemcitabine), purine nucleoside phosphorylase inhibitors (forodesine), adenosine deaminase inhibitors (pentostatin), and alkylating agents (mechlorethamine and chlorambucil), among others (**Table 3**) (60). However, given the limited duration of response and side effect profile associated with chemotherapy, single agent doxorubicin and gemcitabine are typically employed in relapsed or refractory cases of MF/SS. Patients with visceral disease, aggressive non-MF/SS, or those who have failed multiple lines of therapy often receive multi-agent chemotherapy regimens employed for peripheral T cell lymphoma. We will review the most commonly used chemotherapeutic agents.

### 4.1 Alkylating agents

Alkylating agents, such as topical mechlorethamine (nitrogen mustard) and oral chlorambucil and cyclophosphamide, have been used in the treatment of MF for more than 50 years (60, 97, 98). These agents binding to DNA, namely at guanine residues, lead to strand breaks and inter-strand cross-linking, impairing DNA replication (60). Cyclophosphamide, in particular, has been able to induce immunogenic cell death (ICD) (see below). Nitrogen mustards can also generate reactive oxygen species (ROS) (99). Mechlorethamine has been shown to have direct apoptotic effects in malignant skin T cells, but it is not clear if it can cause ICD (100). Historically, mechlorethamine was formulated into aqueous solutions and used as a total body application, which was associated with high rates of delayed-type cutaneous hypersensitivity reactions (≤67%) (101, 102). Ensuing formulations used a petrolatum-based ointment for compounding, which was associated with fewer hypersensitivity reactions (103). While the development of a delayed hypersensitivity reaction limits its prolonged use, it is associated with a better prognosis (101). It is unclear whether the hypersensitivity reaction is itself therapeutic or instead a measure of an intact cell-mediated immunity, as reactivity to the contact allergen 2,4-dinitrochlorobenzene (DNCB) is also associated with better overall prognosis and diminishes with advancing stage of disease (104). It is possible that by inducing a hypersensitivity reaction there is a local increase in T_h_1 cytokines by tumor infiltrating lymphocytes, which could contribute to an anti-tumor response (103). In 2013, the U.S. Food and Drug Administration (FDA) approved a readily available mechlorethamine, 0.02%, gel (Valchor) for the treatment of MF-CTCL. The gel achieved a RR of 58.5% while the ointment achieved a RR of 47.7% in a non-inferiority study compared with mechlorethamine 0.02% compounded in ointment (53). Chlorambucil (2-12 mg daily) has been used in combination with systemic steroids, or in conjunction with ECP, biologic response modifiers, and/or phototherapy in several retrospective cohort studies and case series (51, 60). The main toxicity of chlorambucil, like cyclophosphamide, is myelosuppression (60).

### 4.2 Anthracycline topoisomerase inhibitor

Pegylated liposomal doxorubicin (Doxil) is an anthracycline topoisomerase inhibitor, with direct cytotoxic effects on tumor cells. Interestingly, *in vivo* and *in vitro* studies have found that anthracyclines may also have favorable immune modulatory effects, namely through inducing an immunogenic cell death (105, 106). Physiologic cell death is normally a non-immunogenic event, thereby preventing the development of autoimmunity (107). However, cancer cell death, as induced by radiotherapy or certain chemotherapies, namely anthracyclines, has been shown to promote an anti-tumor response (107). Doxil treated tumor cells, in *in vitro* and *in vivo* studies, are preferentially processed by antigen presenting cells, leading to dendritic cell maturation, and are capable of eliciting a cytotoxic CD8+ T cell response (106). This effect is partly due to release of immune activating molecules called damage associated molecular patterns (DAMPs). This includes translocation of calreticulin (CRT) to the cell surface and release of high mobility group box 1 protein (HMGB1), which both facilitate the recognition and engulfment of dying tumor cells by dendritic cells, *via* binding of TLR4 in the case of HMGB1 (106, 107). In MF/SS, various dosing regimens of Doxil have been evaluated with a RR ranging from 30% to 80% with CR rates of 20% to 60% (52, 108).

### 4.3 Anti-metabolites

Antimetabolites interfere with the cell cycle due to similarities with key cellular substrates or their cognate enzymes. Antimetabolites used for CTCL include gemcitabine, a pyrimidine antagonist, and folic acid antagonists methotrexate and pralatrexate. Gemcitabine is capable of inducing immunologic cell death (109). Methotrexate is a dihydrofolate reductase inhibitor, preventing the conversion of dihydrofolate to tetrahydrofolate, which is required for synthesis of thymidylate and purine nucleotides involved in DNA and RNA synthesis (110). Methotrexate also has immune modulatory functions, which are thought to be a result of inhibition of methionine synthetase and aminoimidazole-carboxamide ribonucleotide transformylase, which ultimately leads to an increase in adenosine (110). Adenosine in turn broadly suppresses effector NK and T cell function, impairs dendritic cell maturation and antigen presentation capacity, and enforces M2 polarization (111). In addition, methotrexate, through an unknown mechanism, is a potent inhibitor of the JAK-STAT pathway, as described above (112, 113). In a monotherapy study of low dose methotrexate (defined as doses <100 mg/week), there was an observed RR of 58% (17 of 29) with a 41% CR in patients with erythrodermic MF and a RR of 33% (20 of 60) with 12% CR in patients with plaque-stage MF (54). Pralatrexate, a novel anti-folate agent, selectively binds and is internalized into cells that express reduced folate carrier type 1 (RFC-1), for which it has a high affinity and is selectively expressed in tumor cells (114). In a dose finding study of pralatrexate in CTCL an overall RR of 45% was observed, with only one CR (55).

## 5 Immune checkpoint inhibitors

Immune checkpoint inhibitors (ICIs) have led to dramatic clinical responses in melanoma, which has attracted the exploration of their use in numerous other cancers, including Hodgkin’s and non-Hodgkin’s lymphomas (115).

### 5.1 Programmed death/programmed death ligand 1 targeting

Programmed death-ligand 1 (PD-L1) can be expressed by hematologic and non-hematologic cells; whereas programmed cell death protein 1 (PD-1), an inhibitory receptor, is expressed following antigen recognition on activated T cells, as well as Tregs, B cells, NK cells and other myeloid populations (116). Canonically, engagement of PD-1 on T cells by its ligand promotes phosphorylation of PD-1 tyrosine residues in the cytoplasmic region, which leads to binding of protein tyrosine phosphatases. This inhibits kinases downstream of T cell receptor (TCR)-CD28 signaling, including PI3K, Ras, extracellular signal-regulated kinase (ERK), and phospholipase C-γ (PLCγ) (116, 117). This results in decreased T cell activation, cytotoxic function, proliferation, survival, and cytokine secretion—enabling immune evasion by tumor cells. In cancer, tumor infiltrating lymphocytes often express high levels of PD-1, characteristic of an exhausted T cell phenotype that is unable to mount an effective anti-tumor response (118). PD-1 inhibition thereby unleashes T cells to affect tumor cell killing. However, the downstream effects of PD-1 inhibition on malignant T cells expressing PD-1 with or without PD-L1 is less clear.

Initial observations in MF/SS found elevated PD-L1 and decreased PD-1 expression on malignant T cells with advancing stages, leading to investigation of ICIs in MF/SS (119). In the phase II study of MF/SS (n=24), the RR was 38% (120). Of the nine responding patients, six had 90% or more improvement by mSWAT. There were no differences between responders and non-responders with respect to pre-treatment PD-1/PD-L1 expression on malignant T cells, IFN-γ/TGF-β gene signatures, or absolute numbers of CD4+ T cells, CD8+ T cells, Tregs, dendritic cells or tumor cells in the TME. However, single-cell spatial phenotyping of the TME from tissue microarrays obtained from skin biopsies of trial patients pre- and on-treatment revealed that responders compared to non-responders had cellular neighborhoods enriched for tumor with dendritic cells, tumor with CD4+ T cells, and decreased zones with Tregs (121). Among responders following PD-1 blockade, increased numbers of activated (ICOS+), proliferating (Ki-67+) CD4+ T cells with cytotoxic function (GZMB+), and CXCL13 expressing tumor cells were noted. Non-responders had greater Treg enriched neighborhoods, including greater numbers of activated (ICOS+) Tregs and a suppressive subset (IDO-1+) of CD8+ T cells. Importantly, they found that a simple metric, termed the *SpatialScore*, which calculates the physical distance ratio of each CD4+ T cell and its nearest tumor cell (“right” distance) relative to its nearest Treg (“left” distance), calculated on a per-cell basis, was predictive of responders versus non-responders pre-treatment. Among SS patients, responders were found to have lower levels of KIR3DL2 expression in malignant cells, which inhibits activation-induced cell death, pre-treatment. And following PD-1 blockade, responders had expanded peripheral blood CD8+ terminal effector and CD8+ effector memory T cell populations (122). Favorable responses to PD-1 inhibition have also been observed in MF cases with PD-L1 structural variants (123). Rare PD-L1 structural variants have been identified in some MF patients (20, 124), similar to PD-L1 3’ untranslated region disruptions reported in extranodal natural killer/T cell lymphomas and adult T cell leukemia lymphoma (125, 126). Disruption of PD-L1 3’ untranslated region in mice demonstrated elevated PD-L1 expression with immune evasion of E.G7-OVA T lymphoblast tumor cells (126). Use of anti-PD-L1 antibody in these mice promoted tumor regression and restored CD8+ cytotoxic T lymphocyte function.

Enthusiasm for ICIs in T cell lymphoma, however, is attenuated by the risk for disease hyperprogression through disinhibition of PD-1 expressing malignant T cells. In T cell lymphomas, PD-1 has been shown to act as a tumor suppressor *via* enhanced PTEN expression and decreased PI3K-Akt signaling (127). Risk of hyperprogression is likely diminished in MF/SS with mono- or bi-allelic loss of *PDCD1*, encoding PD-1 (120). Hyperprogression has been reported in peripheral T cell lymphoma (PTCL) and adult T cell leukemia/lymphoma (128, 129). In a phase II study of pembrolizumab in MF/SS a flare of erythroderma was noted exclusively among SS patients, which correlated with PD-1 tumor expression but did not prohibit continuation of ICI and did not correlate with disease responses or progression (120). While increased proliferation of Sézary cells has been reported following exposure to nivolumab *in vitro* (130), there was no increased percentages of proliferating Sézary cells among non-responders in the phase II trial of pembrolizumab (120).

ICIs are being investigated in combination with targeted therapies. A phase I/II study of nine patients with refractory and advanced CTCL using the PD-L1 inhibitor durvalumab with lenalidomide demonstrated clinical activity (21). Seven (78%) achieved a response with two having 90% or more improvement in mSWAT. The phase II portion is ongoing (NCT03011814). Given the importance of oncogenic PI3K signaling in *PDCD1* deficient malignant T cells, the addition of PI3K inhibitors may mitigate the risk of hyperprogression in T cell lymphomas with PD-1 expression following PD-1 inhibition (NCT04652960). Furthermore, inhibition of PI3K signaling in tumor-associate myeloid cells (see PI3K inhibitor section) can improve responses to ICI in murine models (131). Lastly, nivolumab is being combined with brentuximab vedotin (discussed below), which has been shown to decrease highly activated/suppressive IRF4+ effector Tregs and tumor associated macrophages (132).

### 5.2 Cytotoxic T-lymphocyte-associated protein 4 targeting

Another common checkpoint target, CTLA-4, has had more limited exploration in CTCL. CTLA-4 is expressed soon after T cell activation, primarily in lymph nodes (priming phase), to regulate proliferation and can induce T cell anergy (133). This contrasts with PD-1, which inhibits T cells later in the immune response and primarily expressed in T cells in the peripheral tissue (peripheral tolerance) (133). CTLA-4 is also highly expressed on Tregs, where it is believed to increase Treg immunosuppressive function (134, 135). Therefore, CTLA-4 blockade is thought to increase the activity of anti-tumor effector T cells and inhibit Tregs. However, in clinical trials the impact of ipilimumab on Tregs is less clear, with some reports of increased levels of Tregs following treatment, and other studies showing decreased intra-tumoral Tregs (136, 137). In pre-clinical studies, ipilimumab was able to deplete Tregs *via* antibody-dependent cellular cytotoxicity (ADCC) and phagocytosis (138). Two patients with MF/SS treated with ipilimumab have been reported. A patient receiving the CTLA-4 inhibitor ipilimumab for his advanced melanoma achieved complete MF regression, and another patient with SS treated with ipilimumab experienced rapid clinical response (139, 140). The patient with SS had a highly expressed CTLA-4 and CD28 gene fusion, a putative oncogenic driver. Jurkat cells engineered with this fusion construct proliferated faster than both cells with empty vector and cells with CTLA-4 overexpression (124). It is not clear if ipilimumab efficacy is limited to patients with this specific CTLA-4 fusion protein.

### 5.3 Cluster of differentiation 47 inhibitor

Another potential checkpoint target involves cluster of differentiation 47 (CD47). This transmembrane protein binds to signal regulatory protein alpha (SIRPα), leading to inhibition of macrophage phagocytosis (141). Tumor cells commonly overexpress CD47 to exploit this innate macrophage checkpoint. Therapeutic TTI-621 has a CD47 binding domain of SIRPα that is also associated with the Fc region of human IgG1 (22, 142). Therefore, this immune checkpoint inhibitor not only impairs anti-phagocytic signals but also promotes phagocytic signals through immunoglobulin G1 (IgG1) Fc linking with macrophage Fcγ receptors. A phase I study with TTI-621 intratumoral injections had encouraging results (143). Notably, out of the ten patients with paired assessments, eight (80%) achieved reduction in Composite Assessment of Index Lesion Severity (CAILS) scores in non-injected adjacent lesions. One additional patient had a CAILS score reduction in distal non-injected lesions.

## 6 Radiation

### 6.1 Ionizing radiation

Radiation is one of the oldest modalities of cancer therapy for CTCL (144). This skin-directed therapy includes local or total skin electron beam therapy (TSEBT) and brachytherapy. Brachytherapy is the administration of radiation through flexible catheters that can be secured within fully customized surface molds used for complex curved surfaces, such as the face (145). Ionizing radiation is the most effective single therapy for the treatment of MF, partly due to the unusually high radiosensitivity of malignant T cells (**Table 2**) (29–33, 144). Most often, ionizing radiation induces double-stranded DNA breaks leading to programmed cell death (146). Importantly, radiation can induce an immunogenic cell death. In addition, there is upregulation of MHC class I with tumor antigen peptides on tumor cells, leading to enhanced recognition of tumor cells by cytotoxic T lymphocytes (147, 148). Interestingly, TSEBT has also been used in patients with SS with improvement in leukemic disease in addition to the skin. A case series of three SS patients receiving low-dose (8-12 Gray) TSEBT while on stable combination therapy (interferon, bexarotene) saw a decrease in the percentage of Sézary cells in the blood along with decreased numbers of exhausted T cells and increased IFN- γ secretion by peripheral blood monocytes (PBMCs) (149). The ability of a skin-directed therapy to influence immune parameters in the blood, as well as decrease leukemic cells, may in part reflect generation of a systemic anti-tumor immune response.

Due to the immunomodulatory effects of radiation, “combination therapy” consisting of radiotherapy and immune checkpoint inhibitors may therefore induce long-term immune-mediated antitumor activity (148, 150). Several trials are currently underway to evaluate the safety and efficacy of combining TSEBT with ICI for the treatment of MF/SS (NCT03385226). Additionally, there is a trial combining TSEBT with mogamulizumab (see below), which requires NK cell mediated cellular toxicity for effective tumor cell killing (NCT04128072).

### 6.2 Non-ionizing radiation

Non-ionizing radiation, namely phototherapy, can have anti-proliferative and pro-apoptotic effects on T cells but can also drive favorable and unfavorable inflammatory response through several mechanisms. Ultraviolet radiation (UVR) is absorbed by chromophores in the skin (151). This includes trans-urocanic acid in the stratum corneum, cell membrane lipids, intracytoplasmic molecules such as melanin or amino acids, or most importantly by DNA nucleotides. UVR can cause apoptosis by direct chemical modification of DNA nucleotides or *via* phototoxic products that generate free radicals, reactive oxygen and nitrogen species leading to oxidative damage of DNA (151). It is unclear how the immune effects of ultraviolet A (UVA) and ultraviolet B (UVB) differ. However, a notable difference is that the wavelength of UVR determines the depth of radiation. The UVA spectrum penetrates the deepest and is between 320 and 400nm, whereas the UVB spectrum is between 280-320nm and penetrates only to the superficial dermis (152). UVB may be delivered as full-spectrum (broad-band UVB lamps 270-350nm, along with the shorter wavelengths from UVA spectrum) or delivered as small-spectrum (narrow-band UVB lamps 311-313nm). Narrow-band UVB (nbUVB) therapy was developed as a less harmful alternative to broad-band UVB and to photochemotherapy, both of which have significant side effects and carry a risk of carcinogenesis (153).

#### 6.2.1 Narrowband UVB

nbUVB radiation works by creation of DNA photoproducts, namely pyrimidine dimers, which leads to replication arrest or apoptosis. Its efficacy in MF is likely due to its ability to induce apoptosis in T cells (154). Many studies evaluating the effects of UVB photobiology have been carried out following irradiation of immune cells or keratinocytes *in vitro* or following UVR of murine models (155). In these settings, both release of pro-inflammatory and immunosuppressive cytokines have been observed following irradiation. However, the majority of studies have observed immunosuppressive effects of nbUVB, including altered antigen presentation, Langerhans cell depletion, decreased activity of NK cells (156, 157), and generation of Tregs (147, 158–161). Despite these negative immune effects, nbUVB is one of the most commonly used and effective skin-directed therapies for MF (**Table 3**) (34).

#### 6.2.2 Psoralen and ultraviolet A

PUVA is photochemotherapy consisting of taking the oral drug psoralen and then exposing the skin to UVA light. Psoralen forms DNA crosslinks following UVA exposure leading to DNA damage and apoptosis (162). Unlike nbUVB, the effect of PUVA has been studied in detail in an MF cohort leading to several major observations (163). The first is that PUVA can indeed reduce or eliminate malignant T cell as well as decrease benign T cells from the skin. However, this largely occurred in those with a “low” pre-treatment tumor burden (<10% tumor clone frequency, TCF, the percent of T cells that are malignant). In those with a “high” initial tumor burden (>20% TCF), clinical resolution of inflammation was independent of changes in total malignant or benign T cell numbers but due to a change in benign T cell populations based on characterization of the T cell repertoire by high throughout sequencing (HTS) of the T cell receptor (TCR). Bulk RNA sequencing coupled with this HTS TCR data suggested that PUVA may induce a turnover of benign T cell from T_h_2 to T_h_1 populations. Interestingly, a subset of benign T cells recruited post-PUVA treatment showed markers of TCR-dependent, antigen-specific activation-markers of anti-tumor T cells. Therefore, PUVA may lead to clinical responses by direct clearance of malignant T cells or indirectly *via* generation of an anti-tumor immune response. And, surprisingly, PUVA can lead to clinical responses through clearance of inflammation-producing benign T cells without reducing malignant T cells. Despite the later observation, PUVA has been successfully used in MF with long-term remissions possible (**Table 3**) (35, 164, 165). A number of retrospective cohort studies have evaluated PUVA in combination with IFN-α, but given the lack of a comparator arm it is difficult to assess the full benefit of the addition (166–171). In one randomized clinical trial comparing IFN-α and PUVA versus PUVA monotherapy in CTCL stages I and II, there was not a statistically significant difference in complete remission, but the combination therapy had increased progression-free time (172).

#### 6.2.3 Extracorporeal photopheresis

ECP involves *ex vivo* treatment of a patient’s blood followed by retransfusion into the patient (173). ECP, typically used for MF/SS patients with a low blood burden of disease (**Table 3**) (25, 36), exposes peripheral blood (buffy coat) to UVA radiation following photosensitization with 8-methoxypsoralen (8-MOP) (173). It has direct pro-apoptotic as well as immune modulating effects (174). During the ECP process, platelets adherent to the ECP plate, inadvertently coated with fibrin, engage monocytes and induce them to mature into dendritic cells capable of presenting tumor antigens to generate an anti-tumor immune response (175). While the type of apoptosis induced in leukemic malignant T cells has not been further evaluated, in 8-MOP treated melanoma cell lines, photoactivation induced an immunogenic cell death (176). While ECP has been shown to eliminate alloreactive T cells in GVHD, it has also been reported to induce the generation of Tregs, a potentially unfavorable immune response in CTCL (177). Understanding the effect of ECP on Treg populations in CTCL in the past has been challenging given the difficulty of distinguishing malignant T cells expressing Treg markers from benign Tregs (178). Additionally, in murine models, ECP impairs effector T cell functions (179). Several retrospective and some prospective cohort studies have suggested that combination of interferon or bexarotene with ECP may lead to improved responses, although further studies are needed (60).

#### 6.2.4 Photodynamic therapy

PDT involves the combination of a photosensitizing compound, such as porphyrins (e.g. 5-aminolevulinic acid, ALA) or non-porphyrins (e.g. hypercin), with administration of light of a certain wavelength (180, 181). These photosensitizers may preferentially accumulate in the mitochondria, lysosomes, endoplasmic reticulum (ER), Golgi apparatus, and/or plasma membrane. The light then activates the sensitizer, generating reactive oxygen species (ROS) (182). ROS can be generated by physiologic signaling pathways in T cells leading to activation, differentiation, proliferation, or apoptosis (183). However, high levels of ROS generated by photosensitizers, especially those leading to ER stress, potently induce immunogenic cell death (181). Following PDT, pro-inflammatory cytokines IL-1 and IL-6 are also increased (184). While inflammatory apoptosis of malignant T cells is favorable, Tregs are not as suspectable to ROS (185). In addition, ROS can cause hyporesponsiveness of effector T cells and NK cells (185). Therefore, generation of ROS can be a double-edged sword.

PDT has shown promise for the treatment of MF. Case series evaluating the use of PDT date to the 1990’s (186). Initial studies used ALA, but this was later replaced by methyl-aminolevulinate (MAL), which is thought to have better specificity given its lipophilic properties (186). Despite encouraging responses (**Table 3**) (26), the use of in office PDT for MF is impractical given the size of lamp and need for frequent treatments. Not surprisingly PDT in the past was mainly investigated in unilesional MF (186). Recently, a non-porphyrin photosensitizer, synthetic hypercin (SGX301), which is activated by visible fluorescent light in the 500-650mn range (yellow-red spectrum) has completed its pivotal phase 3 clinical study. In *in vitro* studies activated hypercidin caused greater apoptosis of malignant T cells over benign T cells (187). Notably, hypercidin is a potent inducer of immunogenic cell death (181).

## 7 Targeted therapies

### 7.1 Fusion peptides and proteins

#### 7.1.1 E7777

The high affinity IL-2R (CD25) is overexpressed in the malignant T cells in CTCL (188). E7777 (formerly known as denileukin diftitox) is an engineered fusion protein combining IL-2 and the diphtheria toxin and binds to cells expressing the high affinity IL-2R, including Tregs and malignant T cells, causing endocytosis of the fusion protein (189). After endocytosis, ADP-ribosyltransferase activity of diphtheria toxin inhibits protein synthesis and causes cell death. Therefore, this therapy simultaneously decreases tumor burden and theoretically boosts the anti-tumor immune response by depleting immunosuppressive Tregs (190, 191). However, treatment has been shown to only target activated T cells, and thus resting Tregs remain in circulation (192). Furthermore, this treatment impairs dendritic cell maturation *in vitro* and *in vivo*, creating a tolerogenic dendritic cell phenotype, which induces T cell anergy instead of activated anti-tumor T cells (192). After being approved by the FDA in 1999, denileukin diftitox was taken off the market voluntarily in 2014 in order to create a more purified form with improved bioactivity to reduce the risk for systemic capillary leak syndrome (193, 194). E7777, a purified and reformulated denileukin diftitox, achieved a RR of 32% (six of 19 with PR and zero with CR) in patients with relapsed or refractory CTCL in a phase II trial (46). E7777 has also been investigated in a recently completed Phase III trial (clinicaltrials.gov identifier: NCT01871727), and application for FDA approval within the next year is anticipated after results are published. Resimmune, a similar fusion protein carrying cytotoxic diphtheria toxin, targets cells expressing the CD3ε surface molecule, which is found predominantly in T cells (195). In an inter-patient dose escalation trial in CTCL there was a 36% RR (nine out of 25) with four CRs (196). The modest response rates may be explained by its complex immune modulating function.

#### 7.1.2 Selective γ-chain cytokine inhibitor

BNZ-1 an antagonist pegylated peptide that binds to the common gamma chain signaling receptor for certain cytokines, namely IL-2, IL-9, and IL-15, was investigated in MF/SS (45). It was hypothesized that inhibition of IL-2 and IL-15 would impair cytokine-driven tumor cell survival, inhibition of IL-2 and IL-9 would increase the anti-tumor immune response by decreasing Treg activity, and inhibition of IL-15 would create a beneficial anti-inflammatory effect (45). IL-15 is a putative autocrine survival factor found in elevated levels in MF skin samples and expressed by MF/SS cell lines (197). A phase I/II study of 19 patients with refractory MF/SS demonstrated positive results with 11 participants achieving PR and one CR; a phase 3 trial is being planned (45).

### 7.2 Monoclonal antibodies

#### 7.2.1 Anti-C-C chemokine receptor 4 antibody

Mogamulizumab is a defucosylated, humanized, anti-CCR4 monoclonal antibody. CCR4 is the receptor for thymus- and activation-regulated chemokine (TARC/CCL17) and MDC/CCL22, a predominantly macrophage-derived chemokine (198). As mentioned, CCR4 is mainly expressed on CD4+ T cells (particularly memory T cells), CD4+CD25+FOXP3+ Treg cells, and only on a minority of CD8+ T cells (66). It is a putative marker for T cells with skin homing abilities, as it is thought to play a role in transendothelial diapedesis and epidermotropism (66). Not surprisingly, CCR4 is overexpressed in malignancies of skin homing T cells including MF, SS, and some peripheral T cell lymphomas (199). Mogamulizumab binds to the N terminal domain of CCR4, eliciting tumor cell killing *via* antibody-dependent cellular cytotoxicity (200). Defucosylation of the antibody’s Fc portion enhances binding-affinity of the Fc receptor on effector cells leading to more potent antibody dependent cellular cytotoxicity (ADCC).

In 2018, mogamulizumab was approved for relapsed or refractory MF/SS following a phase III clinical trial of 370 MF/SS patients, comparing mogamulizumab to vorinostat (50). Mogamulizumab achieved a global response of 35% compared to 6% in patients receiving vorinostat. The mogamulizumab group also had longer progression-free survival (median 7.7 months) compared to the vorinostat group (median 3.1 months). Infusion-related reactions, lymphopenia, drug rash [now termed mogamulizumab-associated rash (MAR)], diarrhea, and fatigue were the most common adverse events of any grade in patients receiving mogamulizumab (201). Immune-related adverse events, such as polymyositis, myocarditis, and hepatitis have also been observed, thought to be driven by Treg depletion (201). Recent retrospective cohorts have affirmed a high incidence of MAR in patients with CTCL (17 of 24, 68%) and found that it is associated with clinical responses, leading to the hypothesis that those with effective decreases in Tregs are more likely to experience MAR and achieve a clinical response (202). Patients who experience MAR and/or other immune related adverse events reportedly have higher rates of CR and PR as well as prolonged remissions of disease, in some cases over two years, giving credence to the theory that remodeling of the immune microenvironment contributes to short and long-term therapeutic efficacy (202, 203).

From the phase I/II study, mogamulizumab was observed to have a favorable effect on patients’ immune profile by reducing Treg cells and increasing NK cell killing function but no effect on the absolute number of NK and CD8+ T cell populations were noted at one month (204). However, a real world SS cohort found that mogamulizumab, in addition to depleting benign CD4+ T cells, Tregs, and malignant T cells, also decreased CD8+ T cells and NK cells at one month of treatment. Longer-term follow-up in this cohort revealed the emergence of “immune restoration” involving expansion of naïve and stem memory CD4+ subsets with almost complete disappearance of pre-treatment exhausted lymphocytes (PD-1+ or TIGIT+ CD4+ T cells) and activated Tregs (205). In skin biopsies taken from patients with MAR, an increase in interferon-stimulated gene IFI44L and macrophages expressing CXCL11 and CXCL9 coupled with expansion of new T cell clones expressing CXCR3 (the cognate receptor for CXCL9/11) and genes important for cytotoxic effector function was observed, shedding new insight on the potential role tumor associated macrophages may play in generating prolonged remissions from mogamulizumab (206). Despite favorable changes in the immune environment, disease relapse in the SS cohort appeared to be tied to the emergence of CCR4- malignant T cells (205).

#### 7.2.2 Anti-CD52 antibody

Alemtuzumab, a monoclonal antibody, targets cells expressing CD52, which is expressed on T cells, B cells, monocytes, eosinophils, and a subset of dendritic cells (60). Alemtuzumab is primarily utilized for leukemic CTCL given its limited efficacy in clearing cutaneous disease. This is likely due to its primary mechanism of action, ADCC, which requires NK cells and neutrophils; these are present predominantly in the blood but not in the skin (207). Administration of alemtuzumab causes a depletion of lymphocytes, with depletion of Tregs and exhausted T cells being a favorable immune modulatory effect in CTCL (208). However, CD52 is broadly expressed in effector cells of the innate and adaptive immune response, important for anti-tumor immune responses and protection against infections (60). The overall RR in alemtuzumab is 55% with 32% of patients achieving a CR (47), though RR may be higher in select populations such as SS. Lower doses of alemtuzumab and anti-microbial prophylaxis have prevented many of the infectious complications seen in the earlier studies (47, 207, 209).

#### 7.2.3 Anti-killer cell immunoglobulin-like receptor 3DL2 antibody

Lacutamab is a first-in-class humanized monoclonal antibody targeting inhibitory killer cell immunoglobulin-like receptor (KIR) KIR3DL2, which is aberrantly expressed in MF/SS and minor subpopulations of NK cells, CD8+ T Cells, and CD4+ T Cells (49, 210). In NK cells, KIR3DL2 is one of several inhibitory KIR receptors that recognizes MHC class I molecules (211, 212). Loss of MHC-I in tumor cells or decreased expression of inhibitory KIRs on NK cells leads to activation of NK cell-mediated killing. In MF/SS its function is less clear, but it appears to function as an inhibitory coreceptor—downmodulating CD3-dependent early signaling events following antigen recognition (213). Therefore, it is suspected that KIR3DL2 may protect malignant T cells from activation-induced cell death (AICD) (213). In pre-clinical studies of SS, lacutamab exerted an anti-tumor effect *via* ADCC, rather than through modulation of KIR signaling in malignant cells. A phase I clinical trial of 44 patients with relapsed or refractory cutaneous T cell lymphoma demonstrated 36% RR, predominantly in SS patients (49). Three patients experienced lymphopenia, which was the most common grade 3 or worse adverse event. The effect of lacutamab on NK cells in CTCL cohorts has not been evaluated. However, use of a related antibody directed against KIR2D inhibitory receptors (IPH2101) in multiple myeloma patients found unexpectedly that IPH2101 decreased the responsiveness of the NK cell. A phase II trial investigating lacutamab alone or in combination with chemotherapy in patients with advanced T cell lymphoma is underway (clinicaltrials.gov identifier: NCT03902184). Combination of lacutamab with PD-1 inhibition has been proposed, as stated above, given that SS responders to PD-1 inhibition had lower levels of KIR3DL2 (122).

#### 7.2.4 CD30 antibody-drug conjugate

Brentuximab vedotin (BV) is an antibody-drug conjugate, consisting of the chimeric monoclonal antibody to CD30 linked to monomethyl auristatin E (MMAE), a potent anti-tubulin agent. It was approved by the FDA for CD30+ cutaneous lymphomas in 2017 (214, 215). Upon binding to CD30, the antibody-drug conjugate is internalized, and MMAE is released causing G2/M arrest and apoptosis (216). For this reason, it is used to treat CD30+ lymphoproliferative diseases, including patients with large cell transformation of MF, which often express CD30 on tumor cells (217). Early clinical trials with 28 MF patients showed a RR of 54% with 7% achieving a CR (218). A median duration of response was 32 weeks. This response was not correlated with CD30 expression. Kim et al. evaluated the use of BV in MF with variable CD30 expression and found an overall response rate of 70% with 20 PR and one CR; they found that median CD30_max_ was higher in responders (13%) versus non-responders (3%) and least effective in cases with a CD30_max_ of <5% (219). This study suggested that BV can be effective in MF even with relatively low to intermediate levels of CD30 (CD30_max _>5%). This may be attributable to the additional favorable immune modulatory effects, including depleting immunosuppressive M2 type tumor associated macrophages, inducing dendritic cell maturation, and promoting bystander cell death upon releasing MMAE (even among CD30- tumor cells) (214, 219). Further, BV was shown to deplete immunosuppressive inducible and primary CD30+ Tregs in a dose dependent manner *in vitro* and *in vivo* (132, 220). CD30+ CD8+ T cells, which play a role in anti-tumor immunity, however, were not affected by BV (132, 220). In a phase III clinical trial of 131 patients with CD30+ MF or primary cutaneous anaplastic large-cell lymphoma, BV was compared to physician’s choice of methotrexate or bexarotene (48). Among 48 MF patients, 50% achieved an objective global response lasting at least four months, outperforming the physician’s choice group.

### 7.3 Small molecules

#### 7.3.1 B cell lymphoma 2 inhibitor

The BCL-2 family consists of pro-apoptotic and pro-survival proteins. Shifts in balance between these two protein groups lead to cell death or survival (221). Overexpression of pro-survival BCL-2 proteins allows for various cellular stresses to occur that would otherwise promote apoptosis in non-cancerous cells, and overexpression of BCL-2 in cancers is associated with resistance to therapy (222–224). Likewise, BCL-2 expression is observed in CTCL lines that are sensitive to venetoclax, a BCL-2 inhibitor already approved by the FDA to treat chronic lymphocytic leukemia, small lymphocytic lymphoma, and acute myeloid lymphoma (225–227). An investigator-initiated, single-arm, open label study of venetoclax closed after enrollment of four patients (clinicaltrials.gov identifier: NCT04171791). A single case was published of a SS participant with skin and blood involvement treated with venetoclax who achieved a near CR (228). Despite no immediate plans for additional trials of venetoclax in CTCL, this agent may be best considered as part of a combination therapy to sensitize cells to apoptosis without impeding immune cell function (227). Control of apoptosis is an important mechanism regulating immune cell development and response (229). Despite decreases in T cell counts with venetoclax, largely due to reductions in naïve T cells, antigen-stimulated effector T cells can survive and proliferate *via* upregulation of other pro-survival proteins, such as BCL-xL (229, 230).

#### 7.3.2 Enhancer of zeste homolog 2 inhibitors

EZH2 is the enzymatic component of the polycomb repressive complex 2 (PRC2). PRC2 catalyzes trimethylation of lysine 27 of histone H3 (H3K27me3), which is deposited at CpG-dense promoters and is involved in gene transcriptional repression (231–233). EZH2 represses expression of tumor suppressor genes causing cell cycle progression and cell proliferation. Gain-of-function mutations in EZH2 have been associated with a variety of cancers, including T cell lymphoproliferative disorders (234–237). Additionally, there is a growing body of literature investigating the function EZH2 in the immune system, with many immune cells expressing EZH2 (238). Tumor infiltrating Tregs, for instance, appear to have EZH2 activity. Disruption of EZH2 activity in Tregs promotes pro-inflammatory functions and increases recruitment and function of CD8+ and CD4+ effector T cells (239). Inhibition of EZH2 in NK cells improves NK cell function (240). However, inhibition of EZH2 can impair effector T cell function and dendritic cell antigen presentation (240, 241). Currently, valemetostat (DS-3201b) a highly specific inhibition of EZH2 and EZH1 is being evaluated in a phase II trial for patients with relapsed or refractory peripheral T cell lymphoma, including non-MF/SS CTCL (NCT04703192). A phase I study of 15 patients with relapsed or refractory non-Hodgkin lymphomas, including PTCL, treated with valemetostat had a 53% RR (seven PR, one CR) (242, 243). Among the five patients with T cell lymphoma the RR was 80% (three PR, one CR).

#### 7.3.3 Histone deacetylase inhibitors

There are 18 histone deacetylases (HDACs), which are categorized into four major classes: class I (HDAC1, 2, 3 and 8), class II (HDAC4, 5, 6, 7, 9, and 10), class III (aka sirtuins; SIRT1, 2, 3, 4, 5, 6, and 7), and class IV (HDAC11) (244). In general, histone deacetylation induces gene silencing. The clinical utility of HDAC inhibitors (HDACi) may include disruption of the epigenetic balance in malignant cells or activation of tumor suppressors, but other “off-target” effects include acetylation of transcription factors modulating their function. The current HDACi that are approved or under development are “pan-HDAC” inhibitors (inhibit HDACs in Class I, II, or IV), class specific inhibitors for either class I or class II, or inhibitors of a single HDAC protein. For example, vorinostat is a pan-HDACi and romidepsin inhibits class I HDACs. In multiple cancer models HDACi have been shown to affect several pathways, which favorably lead to cell cycle arrest, induction of apoptosis, and inhibition of angiogenesis (245). However, HDACi have pleiotropic immune modulating functions, which may have opposing effects on the anti-tumor response.

The “off-target” effects of HDACs on the immune system are diverse with both desirable and undesirable results. HDACs can have immunosuppressive effects. Several HDACs in class I, II, and III have been shown to inhibit TLR mediated expression of pro-inflammatory genes such as the p40 subunit of IL-12, IFN-β, and NF-κB activation (244). Class I HDACs have also been shown to inhibit MHC II expression, thereby decreasing antigen presentation. HDACs also increase PD-1L expression (246).

HDACs also have a multitude of immune activating functions. Class I HDACs have been shown to be important for activation of STAT1 and STAT2 dependent interferon signaling (247). Class IIa HDACs, notably HDAC7, have been shown to positively regulate TLR mediated expression of pro-inflammatory cytokines in macrophages, cytokines, and associated receptors for cytotoxic T lymphocyte function (248, 249). Class IV HDACs are thought to play a role in promoting antigen presentation and antigen specific T cell responses (250). Therefore, inhibition of these pathways could impair the interferon-mediated immune response to viral, bacterial, and fungal pathogens and have a negative effect on the adaptive immune response. In fact, mice treated with HDACi are more susceptible to bacterial and fungal pathogens (251).

Given the overlapping and opposing actions of HDACs on the immune system it is unclear if selective inhibition of a particular class of HDACs or an individual HDAC protein would be more beneficial in CTCL. HDACi have been shown to increase tumor immunogenicity, have both a positive and negative regulation of the innate and adaptive immune system *via* modulation of TLR and interferon signaling pathways, and divergent effects on Treg function (244). Inhibition of class I HDACs has been shown to decrease Treg populations and increase immunologic cell death *via* upregulation of FAS, FASL and TRAIL. However, pan-HDACi, likely due to inhibition of class II HDACs, have been shown to increase Tregs and their function (252). Resistance may be related to an inability to orchestrate a favorable TME or may be related to properties intrinsic to the tumor cell. At the single cell level, for instance, SS has been shown to have a high degree of heterogeneity with differing sensitivity and resistance to HDACi (253). Clinical trials of romidepsin have shown an overall RR of approximately 30% with 6% of patients achieving CRs (41). As suggested above, infections (58% of patients for romidepsin) were common adverse events (254). Vorinostat and romidepsin have been shown to suppress NK and dendritic cell function in patients with CTCL (255). In a patient with SS that effect could be overcome with addition of an immune activating agent, such as TLR agonists or IFN-α when evaluated *in vitro* (255, 256). Therefore, future rational combination therapies with an immune activating agent and HDACi may synergistically enhance the anti-tumor response and decrease the risk of infections. Vorinostat and romidepsin are both approved for use in CTCL. Additional HDACi have been investigated in CTCL including panobinostat with 20% RR (40), belinostat with 14% RR (257), topical SHAPE gel also known as SHP-141 with interim results of 32% showing mSWAT response at 6 months (258), and resminostat with no published results (259).

#### 7.3.4 Immunomodulator

Lenalidomide is a derivative of thalidomide (260). It binds E3 ligase protein cereblon (CRBN) directing protein ubiquitination and degradation of the IL-2 transcriptional repressors Ikaros (IKZF1) and Aiolos (IKZF3) (260, 261). Through modulation of these transcription factors, and likely others, it is able to have diverse effects on the innate and adaptive immune system. These effects include an increase in antigen presentation capacity, expansion and increased activity of cytotoxic CD8+ T cells, and a decrease in T_h_2 cytokine expression (261). In a phase II study of lenalidomide in refractory MF/SS, there was an overall RR of 28%; all were PR. The median duration of response was 10 months (39). In these patients, while there was an overall decrease in malignant CD4+ T cells and an increase in CD8+ T cells in MF skin lesions, an increase in CD25+ and FOXP3+ staining was also observed (39). This increased T regulatory phenotype may be secondary to the increase in IL-2 induced by lenalidomide.

#### 7.3.5 Janus kinase/signal transducer and activator of transcription inhibitors

The JAK-STAT pathway, which transduces signals from cytokines, interleukins and growth factors, has been shown to be dysregulated in both MF and SS (4, 262). The signaling begins with binding to the cell-surface receptor which results in dimerization and causes activation of JAK tyrosine kinases. Subsequently, activated JAKs phosphorylate specific tyrosine residues on the receptor and act as docking sites for STATs, which are cytoplasmic transcription factors (61). Once STAT proteins bind to the receptor, the JAKs phosphorylate the STAT proteins, which dimerize and translocate to the nucleus to induce expression of genes involved in inflammation. STAT4, which is required for T_h_1 differentiation and is activated downstream of IL-12 signaling, has decreased expression in the skin and blood of patients with advancing CTCL stage (262). STAT5, activated downstream by IL-2, IL-7 and IL-15 signaling, and STAT3, activated downstream by IL-2, IL-6, IL-7, IL-9, IL-10, IL-15 and IL-21, may be constitutively activated independent of cytokines in MF/SS (262–264), although there is conflicting data (265). Constitutively activated STAT3 can increase survival and resistance to apoptosis in malignant T cells by increasing the anti-apoptotic protein BCL-2 (266). Furthermore, activation of STAT3 has been shown to induce expression of immunosuppressive ligand PD-L1 (77). STAT5 induces anti-apoptotic proteins, cell cycle genes (cyclin D and c-myc) and IL-4, which promotes carcinogenesis (61). In addition, its activation induces microRNA-155 that degrades STAT4 further solidifying the T_h_2 bias in the microenvironment (262, 267, 268). Furthermore, STAT3 and 5 are also important for the function of Tregs (269). Interestingly, methotrexate has been found, using a luciferase-based transcriptional assay in a Drosophila model system where a library of 2000 small molecules were screened, to be a potent inhibitor of the JAK-STAT pathway at doses that are equivalent to that seen in patients taking low-dose oral methotrexate (113).

While inhibition of JAK appears to be an attractive therapeutic candidate, malignant T cells do not exist in an isolated environment. As mentioned in the biologic response modifiers section, type I and II interferons activate the JAK-STAT pathway, leading to expression of interferon response genes with anti-proliferative and pro-apoptotic effects on malignant T cells. It is possible that the favorable effects of interferon-associated JAK-STAT signaling are blunted by JAK inhibitors. TLR-stimulated plasmacytoid dendritic cells, known for their ability to produce high levels of IFN-α for instance, had robust inhibition of IFN-α production in response to JAK inhibitor tofacitinib *in vitro* (270). Furthermore, with constitutive activation of STATs downstream from JAK in some patients, there is controversy over the ability of JAK inhibition to overcome downstream activation.

The FDA approved JAK inhibitors, including tofacitinib and ruxolitinib, demonstrated anti-tumor properties in CTCL cell lines (271–273), which has subsequently led to recent clinical trials. In a phase II trial of patients with relapsed or refractory PTCL or CTCL receiving ruxolitinib, a 20% RR was achieved among the five evaluable patients with CTCL (42). A phase IIa study of 37 patients with CTCL and 61 patients with PTCL investigated a small molecule spleen tyrosine kinase (SYK)/JAK kinase inhibitor called cerdulatinib (274). SYK is a tyrosine kinase, with its signaling pathway thought to be an oncogenic driver in multiple cancers, including immature hematopoietic neoplasms with T cell differentiation (275). The RR was 35% in CTCL patients overall. In MF the RR was 45%, with 9% of these participants achieving CR (37). Patients with Sézary syndrome had a 17% RR with no CRs.

#### 7.3.6 Phosphoinositide 3-kinase inhibitors

The PI3K-Akt pathway has been implicated in several cancers and is an important signal transduction pathway for immune cells (276, 277). There are three classes (I-III) of PI3Ks (278). Class I, the most relevant to oncology, consists of four isoforms: α, β, δ, and γ. (271) PI3K γ and PI3Kδ are expressed in hematopoietic cells, but PI3Kδ is the dominant isoform in T cells, mediating downstream signaling of the TCR, costimulatory and cytokine receptors (279). PI3Ks phosphorylate the 3-hydroxyl group of the inositol ring found in phosphatidylinositol lipid substrates (280). The 3-phosphoinositides (PIP_3_) mediate the function of multiple effector proteins that bind these lipids. In turn, PI3Ks play a role in intracellular vesicular transport, cell cycle progression, and cell growth, survival, and migration.

Activating mutations in the PI3K-Akt pathway and constitutive Akt activity have been observed in CTCL, supporting the importance of PI3K signaling for malignant T cell survival and proliferation (38). Duvelisib, a PI3Kγ and PI3Kδ inhibitor, was approved by the FDA in 2018 for refractory chronic lymphocytic leukemia and small lymphocytic leukemia and has demonstrated activity in T cell lymphoma cell lines (281, 282). In a phase 1 study of duvelisib for patients with relapsed or refractory PTCL or CTCL, a RR of 31.6% (six of 19) was observed in the CTCL cohort (38). Of note, PI3K inhibition may also affect the immune microenvironment. In PTCL patient-derived xenografts treated with duvelisib a shift in polarization from immunosuppressive M2 macrophages to an inflammatory M1 phenotype, which promotes anti-tumor responses, was observed (38). In other murine models, P13K inhibition of tumor associated myeloid cells was able to sensitize tumors to ICI (131). However, these murine models lack an intact immune system to understand the full effects of P13K inhibition on the microenvironment. For example, PI3K signaling is important for antigen- and chemokine-dependent effector cell trafficking to peripheral sites of inflammation through regulation of leukocyte function-associated antigen-1 (LFA-1) (279). LFA-1 is needed for transendothelial egress and establishment of immunological synapse with antigen presenting cells (279). Therefore, PI3K inhibition may limit migration of anti-tumor effector T cells to the skin and effective engagement with antigen presenting cells thereby hindering the anti-tumor response. However, treatment of CD8+ T cells *ex vivo* with duvelisib, leads to enhanced T cell activation and cytotoxicity (283). Therefore, the net result of PI3K inhibition is unclear. Tenalisib (RP6530), another dual PI3Kδ and PI3Kγ inhibitor, is also under investigation, with a recent phase I/Ib study of relapsed or refractory PTCL and CTCL and RR of 45% was observed (43). A phase I/II study evaluating tenalisib in combination with romidepsin was recently completed (NCT03770000). Duvelisib was also investigated in combination with romidepsin or bortezomib in a phase I/II trial of PTCL/CTCL, based on *in vitro* drug screens predicting synergy. The RR in CTCL was 46% with romidepsin and 28% with bortezomib; no CRs were observed in either group (284).

#### 7.3.7 Proteosome inhibitor

Proteasomes are fundamental to the ATP-dependent proteolytic pathway in eukaryotic cells (285). In fact, most cellular protein degradation occurs through proteasome catalyzed degradation (286). The FDA approved the first proteasome inhibitor, bortezomib (PS-341), for multiple myeloma in 2003 (287, 288). Bortezomib is a modified dipeptidyl boronic acid that is a reversible inhibitor of the 26S proteasome specifically (287). Bortezomib has pro-apoptotic and anti-proliferative effects *via* inhibition of NF-kB and induction of the unfolded protein response (289). It has also been shown to increase tumor cell killing by dendritic cells, likely due to induction of immunogenic cell death (290), and by NK cells (291, 292). A phase II trial of 15 patients with MF and unspecified peripheral T cell lymphoma explored the anti-tumor properties of bortezomib (28). Among the 10 patients with MF the RR was 70%, with one CR and six PRs. Responses were remarkably durable, with the CR in remission at the latest follow up at 12 months and with the PRs lasting at least seven months.

#### 7.3.8 Retinoids

Several retinoids have been used in the treatment of CTCL, including etretinate, acitretin, all trans retinoic acid (ATRA), alitretinoin and isotretinoin (293). Retinoids exert their biologic effect *via* binding and activation of the retinoic acid receptor (RAR) isoforms RAR α, β, and γ, which form a heterodimer with each other or with retinoid X receptors (RXRs), or, in the case of isotretinoin, through indirect effects on the RAR signaling pathway (293). They have anti-proliferative, pro-apoptotic effects in CTCL, as well as immune modulating effects (294). Retinoids can induce IFN-γ partly *via* IL-12 (295), upregulate Langerhans cell antigen presentation capacity and coreceptors required for T cell activation (296), and increase NK cell activity (60).

#### 7.3.9 Rexinoid

Bexarotene, a novel rexinoid, is a ligand for the RXR isoforms: RXR α, β, and γ. RXR can form heterodimers with the RAR, vitamin D receptor, thyroid hormone receptor, peroxisome proliferator activator receptor, liver X receptor, and the farnesoid X receptor to induce gene transcription. Bexarotene has anti-proliferative and pro-apoptotic effects on malignant cells (60). It has been shown to downregulate T_h_2 cytokines (297) and limit migration of malignant T cells *via* decreased expression of CCR4 on T cells and decrease of E-selectin on endothelial cells (298). Additionally, bexarotene in MF patients reduces CCL22 production by M2-polarized tumor associated macrophages (299). CCL22, similar to CCL17, can recruit Tregs (65). Bexarotene was also shown in leukemic cell lines to increase expression of IL-2R, which may be beneficial if combined with E7777, which targets IL-2R expressing cells (discussed above) (300). Bexarotene induced an objective RR of 45% with 13% of patients achieving a CR (27). Bexarotene has been used in combination with many other treatments, including PUVA, interferons, ECP, and others, and while this combination therapy appears safe it is unclear if there is appreciable benefit (60, 301).

## 8 Toll-like receptor agonists

The use of toll-like receptor (TLR) agonists for the treatment of MF is a promising new area of investigation. Toll-like receptors are expressed on various immune cells and sense a variety of foreign molecules, sequences, and peptides to trigger innate immune response by increasing the function of antigen presenting cells (APCs) as well as the expression of type I interferons, which promote a T_h_1 immune response (302, 303). Upon stimulation of dendritic cells, as well as NK cells, by agonists of TLR7, TLR8 or TLR9, there is an increase in IFN-α, IL-12 and IL-15 expression and upregulation of costimulatory markers for improved antigen presentation (302–305).

Several TLR agonists have been evaluated over the years, however only imiquimod, a TLR7 agonist, is available clinically. While several case reports have supported the use of imiquimod to treat MF, a randomized placebo-controlled trial of imiquimod failed to show that it was better than a placebo cream (24, 306–311). It is unclear if inherent differences in the host immune system or of the malignant T cells drives this variable clinical response. Most recently, resiquimod gel, a TLR7 and 8 agonist completed a randomized placebo controlled phase II trial, but the results are not yet published (302). In the phase I trial, a notable 92% of patients had a 50% or greater improvement in mSWAT (11). In addition, resiquimod induced regression of untreated lesions in select cases, suggesting an abscopal effect. Responders were found to have increased numbers of benign T cells with increased effector function and increased activation of circulating dendritic cells (11).

In the past, a phase I study of intratumoral injection of a TLR9-activating class B CPG motif oligodeoxynucleotide (ODN) (CPG-7907) in lesions of MF was investigated and a decrease in CD25+FOXP3+ T cells and an increase of plasmacytoid dendritic cells was observed (312), but further clinical development is not planned (19, 313). Interestingly, when CPG-A and CPG-B ODNs were evaluated *ex vivo* on PBMCs of patients with SS in comparison to healthy volunteers, SS PBMCs had dampened pro-inflammatory responses to ODNs, and CPG-A, but not CPG-B, was capable of stimulating NK cell function (313). This may in part explain the poor performance of CPG-7907, but also highlight the importance of combining TLR agonists with other immune activating agents or one with direct cytotoxic ability to overcome the immunosuppressive effects of malignant T cells. Recently, a phase I/II trial investigating the anti-tumor response of poly-ICLC, a TLR3 agonist, in combination with tremelimumab and durvalumab in patients with relapsed, advanced cancers, including cutaneous T cell lymphoma (NCT02643303) has been completed.

## 9 Discussion

Cancer therapies used for the treatment of CTCL, old and new, may affect the immune compartment, whether intended or not. Despite not being the main target, the therapeutic efficacy of certain agents may be aided by favorably remodeling the microenvironment, including boosting anti-tumor effector cells. Advantageous immune effects of therapies used for CTCL include inducing immunogenic cell death (PDT, chemotherapy, radiation), maturation of dendritic cells/antigen presenting cells (lenalidomide, ECP, interferons, and TLR agonists), reduction of immunosuppressive M2 macrophages (BV, duvelisib) or Tregs (mogamulizumab, alemtuzumab, BV), elimination of exhausted T cells (mogamulizumab, pembrolizumab, alemtuzumab), increasing levels of T_h_1 cytokines (interferon, TLR agonists), and recruitment of cytotoxic CD4+ or CD8+ T cells (pembrolizumab), among others. While several agents may cause myelosuppression or lymphopenia, not all T cell populations are affected equally. Such is the case for venetoclax, which decreases naïve T cells but spares effector T cells making it an attractive candidate to combine with therapies that depend on T- or NK- cell cytotoxicity, and mogamulizumab which preferentially depletes CCR4+ T cells, including Treg cells. We also eagerly await the results of several novel drugs under investigation, summarized in **Table 5**. While we are not certain what will be the most effective therapeutic regimen, the current trend appears to be combination therapy based on the effects of tumor cell and microenvironment. An ideal combination therapy would debulk disease *via* effective and immunogenic killing of highly immunosuppressive tumor cells without causing broad immunosuppression, coupled with activation and education of the immune system to generate a potent anti-tumor immune response for long-term disease remission.

**Table 5:** Therapies under investigation for the treatment of CTCL.

Drug	Mechanism of Action	Route	Clinical trial phase	NCT number	Status
**Monotherapy**					
Resiquimod	TLR7/8 agonist	Topical	Phase II	NCT03292406	Completed, no published results
SHP-14	HDAC inhibitor	Topical	Phase II	NCT02213861	
Venetoclax	BCL-2 inhibitor	PO	Phase I	NCT04171791	
Atezolizumab	PD-L1 inhibitor	IV	Phase II	NCT03357224	Active
Resminostat	HDAC inhibitor	PO	Phase II	NCT02953301	
Valemetostat Tosylate	Dual EZH1/EZH2 inhibitor	PO	Phase II	NCT04703192	
ATLCAR.CD30. CCR4 / ATLCAR. CD30 cells	CD30 directed autologous CAR T cells with or without CCR4 expression	IV	Phase I	NCT03602157	Recruiting
LB1901	CD4 directed autologous CAR T cells	IV	Phase I	NCT04712864	
CD37 directed CAR-T	CD37 directed autologous CAR T cells	IV	Phase I	NCT04136275	
Lacutamab	KIR3DL2 inhibitor	IV	Phase II	NCT03902184	
Microneedle array – doxorubicin	Chemotherapy	Topical	Phase I	NCT02192021	
**Combination Therapy**					
Poly-ICLC with tremelimumab and durvalumab	TLR3 agonist; CTLA-4 inhibitor; PD-1 inhibitor	SC; IV; SC	Phase I/II	NCT02643303	Completed, no published results
Duvelisib with romidepsin or bortezomib	PI3K inhibitor; HDAC inhibitor; proteosome inhibitor	PO; IV; SC	Phase I	NCT02783625	Active
Interferon-.γwith pembrolizumab	Interferon; PD-1 inhibitor	SC; IV	Phase II	NCT03063632	
Radiation with pembrolizumab	Radiation; PD-1 inhibitor	IV	Phase II	NCT03385226	Recruiting
Talimogene laherparepvec with nivolumab	Oncolytic virotherapy; PD-1 inhibitor	SC	Phase II	NCT02978625	
Duvelisib with nivolumab	PI3K inhibitor; PD-1 inhibitor	PO, IV	Phase I	NCT04652960	
Durvalumab with lenalidomide	PD-L1 inhibitor; CRBN modifier	IV; PO	Phase II	NCT03011814	
Sintilimab with chidamide	PD-1 inhibitor; HDAC inhibitor	IV; PO	Phase II	NCT04296786	
TSEBT with mogamulizumab	Radiation; anti-CCR4 Ab	IV	Phase II	NCT04256018	
TSEBT with mogamulizumab	Radiation; anti-CCR4 Ab	IV	Phase II	NCT04128072	Not yet recruiting

BCL-2 = B-cell lymphoma 2; CAR = chimeric antigen receptor; CD = cluster of differentiation; CTLA-4 = cytotoxic T-lymphocyte associated protein 4; EZH = enhancer of zeste homolog; HDAC = histone deacetylase; KIR3DL2 = Killer Cell Immunoglobulin Like Receptor, Three Ig Domains And Long Cytoplasmic Tail 2; NCT = National Clinical Trial; PD-1 = programmed cell death protein; PD-L1 = programmed death-ligand 1; PI3K = phosphoinositide 3-kinases; PO = oral; Poly-ICLC = polyinosinic-polycytidylic acid; SC = subcutaneous injection; IV = intravenous; TLR = toll-like receptor

There is a growing body of literature on the immune effects of drugs, but much remains unknown. An inherent challenge in understanding the immune effects of therapies is that they may be solely driven by the selective killing of malignant T cells, which are highly immunosuppressive in CTCL. Regardless, with successful eradication of suppressive malignant T cells, immune restoration is observed across several therapies. And through better understanding of the immune modulating effects of our therapies, superior combination therapies may be developed, and prolonged remissions realized, such as that achieved by allogenic stem cell transplantation.

## Data availability statement

The original contributions presented in the study are included in the article/supplementary material. Further inquiries can be directed to the corresponding author.

## Author contributions

CF prepared the initial draft of the manuscript and participated in revisions. KA created the tables and participated in revisions. NL participated in revision. CL participated in conception of the review, prepared the initial draft, created the figure, and participated in revisions. All authors contributed to the article and approved the submitted version.
